# Developmental riboflavin deficiency results in structural and functional changes in the neural retina and RPE

**DOI:** 10.1016/j.redox.2025.103772

**Published:** 2025-07-16

**Authors:** Xue Zhao, Mustafa S. Makia, Muna I. Naash, Muayyad R. Al-Ubaidi

**Affiliations:** Department of Biomedical Engineering, University of Houston, 3517 Cullen Blvd., Houston, TX, 77204, USA

**Keywords:** Riboflavin, Retbindin, Ariboflavinosis, Retinal degeneration, RPE dystrophy

## Abstract

The retina, a metabolically active tissue, relies on adequate flavin levels for optimal function. Our previous research demonstrated that ablation of RTBDN, a retina-specific riboflavin binding protein, plays a pivotal role in maintaining flavin levels, leading to progressive retinal degeneration. This raises the fundamental question of how riboflavin deficiency impacts retinal structure and function. We have previously evaluated an adult diet-induced model of riboflavin deficiency and showed that lack of flavins resulted in severe functional and structural deficits in the neural retina and retinal pigment epithelium with increased oxidative stress and metabolic dysregulation. Ariboflavinosis resulting from mutations in riboflavin transporters manifests early in life and is treatable with riboflavin supplementation. To mimic ariboflavinosis, we established an early-onset dietary model. At postnatal day 30, we observed a pronounced retinal phenotype characterized by early decline in cone function and subsequent loss of cone photoreceptors, while rods remained unaffected. Notably, RTBDN exhibited a biphasic response to early ariboflavinosis: initially upregulated, suggesting a protective role in maintaining retinal flavin levels, but decreased as deficiency persisted with subsequent photoreceptor functional decline. Riboflavin supplementation partially ameliorated these phenotypes by restoring retinal flavin and RTBDN levels, resulting in improvements in retinal structure and function. However, some cellular changes in the RPE remained irreversible and cone count was not restored. These findings underscore the critical roles of riboflavin and RTBDN in maintaining retinal and RPE health and highlight the importance of early detection and intervention for optimal therapeutic outcomes.

## Graphical abstract

The retina is a highly metabolically active tissue that depends on sufficient flavin levels to function properly. RTBDN, a protein found specifically in the retina, helps maintain these flavin levels by binding to riboflavin (vitamin B2). This raises an important question: how does ariboflavinosis—a condition caused by mutations in flavin transporters—affect the structure and function of the retina?To investigate this, we created an early-onset dietary model to mimic ariboflavinosis. In this model, we observed a clear retinal response: cone function declined early, followed by the loss of cone photoreceptors. Interestingly, RTBDN showed a two-phase reaction to the deficiency. It was initially upregulated, possibly to help preserve flavin levels in the retina, but its expression decreased as the deficiency continued.

**Figure:**
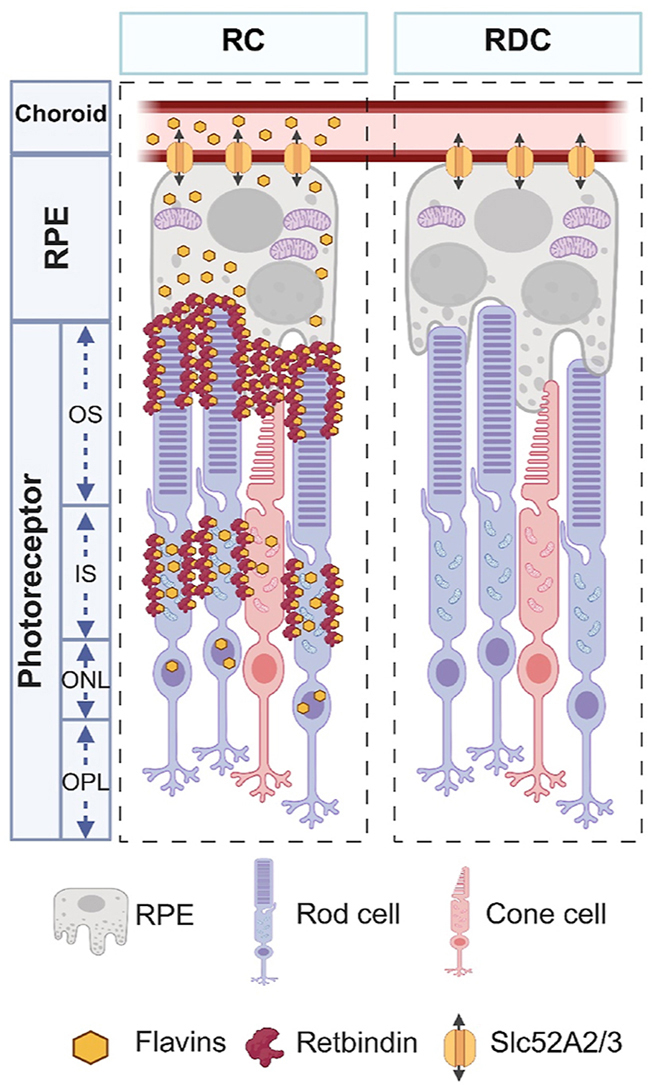

## Abbreviations

ERG: electroretinography
FAD: flavin adenine dinucleotide
FMN: flavin adenine mononucleotide
HPLC: high-pressure liquid chromatography
IPL: inner plexiform layer
IS: inner segments
NAD: nicotinamide adenine dinucleotide
NADP: nicotinamide adenine dinucleotide phosphate
ONL: outer nuclear layer
OPL: outer plexiform layer
S/M Opsins: short/middle wave length opsins
OS: outer segments
PBS: phosphate-buffered saline
PECS: pigment epithelium/choroid/sclera
PLP: pyridoxal 5′-phosphate
RC: regular chow
RDC: riboflavin-deficient chow
RF: riboflavin
RFVT: Riboflavin transporter
RPE: retinal pigment epithelium
RTBDN: retbindin
RTD: Riboflavin transporter deficiency
SLC52A2: riboflavin transporter
SLC52A3: riboflavin transporter

## 1 Introduction

Riboflavin (RF) is a water-soluble vitamin belonging to the B-complex family that plays a critical role in the body's metabolism by contributing to redox homeostasis, oxidative phosphorylation, and other enzymatic reactions [[1], [2], [3], [4], [5], [6]]. RF is obtained from dietary sources such as dairy products, meats, eggs, green vegetables, whole grains and cereals [[4], [5], [6], [7], [8]]. RF is absorbed in the small intestine via RF transporter 3 (RFVT3) [6,9,10]. Once in target cells, RF is transported by either RFVT1 or RFVT2 and subsequently converted into flavin mononucleotide (FMN) by the enzyme RF kinase. FMN is then further converted into flavin adenine dinucleotide (FAD) by FAD synthetase [3,[4], [11]]. Both FMN and FAD are essential cofactors involved in redox reactions, supporting the electron transport chain and enabling energy production through oxidative phosphorylation as well as the metabolism of carbohydrates, proteins, and fats [2,6,9]. The retina, being the most metabolically active tissue, has a high demand for flavins [12,13]. Previously, we demonstrated that retbindin (RTBDN), a retina-specific RF binding protein, plays a key role in maintaining flavin levels in the retina [14,15]. Ablation of *Rtbdn* leads to a significant reduction in retinal flavin levels and causes retinal degeneration [[15], [16], [17]].

Ariboflavinosis (RF deficiency) is a condition that can result from inadequate dietary intake, malabsorption disorders, increased physiological demands, alcoholism, intake of certain medications that may affect riboflavin metabolism or absorption and riboflavin transporter deficiency [6,18,19]. Ariboflavinosis can disrupt metabolic processes, affecting β-oxidation of fatty acids, energy production, oxidative stress, and cellular development [3,4,18,20,21]. However, timely diagnosis of the condition is often lacking, which manifests through neurological and visual symptoms[22].

Previously, we demonstrated the crucial role of RF in adult retinal homeostasis by using an adult-onset ariboflavinosis mouse model [21]. We found that RF deficiency caused the downregulation of retinal flavins, RTBDN levels, and gradually led to functional decline, retinal degeneration, changes in the RPE and abnormalities in retinal metabolic activity [21].

RF transporter deficiency (RTD; also known as Brown-Vialetto-Van Laere syndrome) is a progressive inherited neuropathy with childhood onset, caused by mutations in *SLC52A2* (RTD2) or *SLC52A3* (RTD3), which encode the human RF transporters 2 (RFVT2) and 3 (RFVT3), respectively [2,10,20,23]. The most frequently observed phenotype of RTD is a progressive peripheral and cranial neuropathy characterized by vision loss, deafness, sensory ataxia, muscle weakness, and respiratory compromise [23,[24], [25]].

**Fig. 1:**
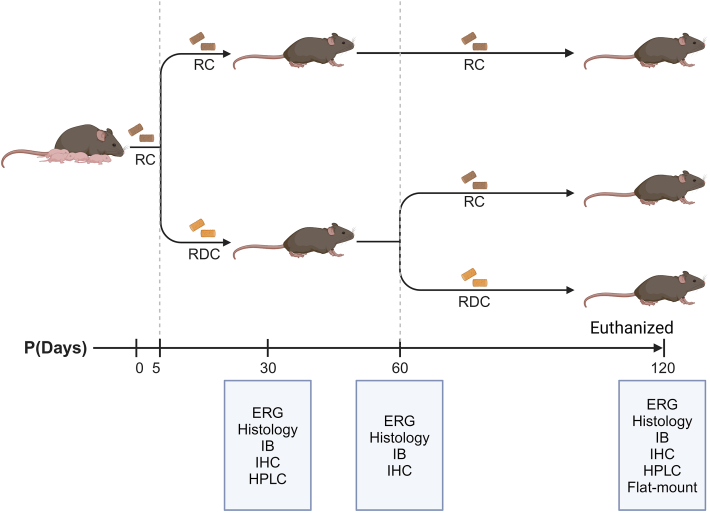
**Early-onset ariboflavinosis mouse model and experimental design.** Wild-type mice and their pups were initially maintained on regular chow (RC, 8 ppm RF). At postnatal day 5 (P5), they were switched to an RF-deficient chow (RDC) until designated time points (P30, P60, P120). At P60, a subset of mice was returned to regular chow (RDC > RC) and maintained until P120. At designated ages, electroretinography (ERG), histology, immunoblotting (IB), immunohistochemistry (IHC), high-performance liquid chromatography (HPLC), and flat-mount were performed to assess retinal function, structure, RPE integrity, RTBDN, and flavins levels, respectively. Mice maintained on regular chow served as controls for all experiments. Figure created with BioRender.com (accessed on Jul 26th, 2024).

Research exploring the effects of RF transporters deficiency showed that *Slc52a3* knockout mice exhibit neonatal lethality, brain hypoplasia, thinner cortical layers, and metabolic disorders caused by insufficient RF supply [26]. Most *Slc52a3* knockout mice died with hyperlipidemia and hypoglycemia within 48 h after birth [27]. Surprisingly, the hypoplastic phenotype was rescued with RF supplementation [26]. Similarly, ablation of *Slc52a2* leads to embryonic lethality [28]. To study a model that mimics RTD while circumventing embryonic lethality and to determine the role RF plays in visual developments, we generated a nutritional model of early onset ariboflavinosis by switching mother and pups at postnatal day (P) 5 from regular chow (RC) to RF deficient chow (RDC) (Fig. 1). We hypothesize that early-onset RF deficiency, during critical periods of retinal development, may impair key processes, leading to permanent structural and functional consequences. Through this model, we aimed to uncover how early RF deficiency impacts cellular development, offering insights into developmental processes disrupted by RF deficiency and its association with retinal dysfunction. We found that the retina and RPE initially retained some flavins, but over time, flavin levels decreased, coinciding with a reduction in RTBDN levels, which were initially upregulated. These changes were accompanied by retinal functional decline, cone cell loss, inner retinal structural changes, and RPE dystrophy. The findings in the present study underscore the importance of RF in maintaining developmental retinal homeostasis.

## 2 Material and methods

### 2.1 Animals

All animal experiments were approved by the University of Houston Institutional Animal Care and Use Committee (IACUC), followed NIH guidelines for the care and use of laboratory animals, and by the Association for Research in Vision and Ophthalmology (ARVO). Prior to experimentation, genotyping confirmed that all mice were negative for the RPE65 Leu450Met variant and the *rd8* allele, and they were on a C57BL/6 J-129S1/SvImJ background. Mice were reared in controlled cyclic light environments with 12/12-h light/dark cycle and an average illumination of around 30 lux. Food and water were provided *ad libtum* with the only variable being the presence or absence of RF in the chow, whereby the regular chow (RC) contained standard RF-supplement (AIN-93G; Envigo, Indianapolis, IN) while RF-Deficient-AIN-93G; (Envigo, Indianapolis, IN) was used as RF-deficient chow (RDC) (Fig. 1). Both sexes were included in the study and exhibited uniform responses to treatment and demonstrated no difficulties in producing viable offspring. The ages of the mice used and the numbers of experimental replicates are stated in the figure legends. To minimize bias, treatment identities remained concealed until completion of all analyses, including histology measurements and ERG evaluations.

### 2.2 Antibodies

Antibodies used are summarized in Table S1. A monoclonal antibody (6B6H5E11) specific to RTBDN was generated using standard hybridoma technology. The specificity of this antibody was confirmed by the absence of the corresponding band in the Rtbdn knockout retinal sample, while a distinct band (∼30 kDa) was observed in WT RC samples. β-actin was used as a loading control.

### 2.3 Tissue collection

Tissue collection for HPLC and immunoblots was consistent with established methodologies detailed in prior literature [15,17,29,30]. Briefly, the neural retina (retina, herein), the rest of the eyecup consisting of RPE, choroid, and sclera (PECS) were collected and immediately frozen in liquid nitrogen and stored at −80 °C before processing. For immunofluorescence, eyes were enucleated, incubated in modified Davidson fixative (33 % ethanol, 11 % acetic acid, and 9 % paraformaldehyde) for 3–4 h. To prepare RPE flat mounts, whole eyes were collected, and a puncture was made at the cornea, and fixed in 4 % paraformaldehyde for 2 h. After fixation, the cornea was removed and carefully teased out the retina from the eye cup, leaving the RPE, choroid, and sclera behind. Gently and equally cut the eye cup into four pieces and store in 1 × PBS at 4 °C until used.

### 2.4 High-performance liquid chromatography (HPLC)

The process for the micro-extraction of flavins from tissue was as published previously [29]. In summary, frozen tissues were homogenized using a handheld motorized pestle in 100 μl phosphate-buffered saline solution (1 × PBS, pH 5.1, unless specified otherwise). From the resultant mixture, a 30 μl aliquot was reserved for determining protein concentration. The rest was heated up at 80 °C for 30 s, cooled down on ice, and then treated with 10 % trichloroacetic acid at room temperature for 15 min to precipitate proteins. The mixture was centrifuged at 14,000×*g* for 10 min, supernatant collected and filtered through a 0.45 μm filter in preparation for HPLC analysis. HPLC analysis was performed immediately after extraction to avoid any effect on flavin levels due to repeated freezing and thawing. The chromatography was conducted in accordance with prior descriptions [29]. To separate and detect flavins by using a gradient method, mix solution A (50 mM phosphate buffer, pH 3.0) and solution B (100 % acetonitrile) at a flow rate of 0.8 ml/min. The Waters HPLC system (Water Corporation, Milford, MA, USA) is comprised of a binary pump 1525, an auto-sampler (2707), a multi-wavelength fluorescence detector (2475), and an X-Bridge C18 3.5 μm column with dimensions of 4.6 mmΧ250mm. The flavin quantification followed an established HPLC methodology [29].

### 2.5 Histology and morphometric analysis

Histologic examination and morphometric analyses were performed as described previously [15,17,21]. To maintain orientation, the superior cornea was cauterized with a hot needle before the careful dissection of the eyes. Eyes were fixed for 3–4 h at 4 °C in modified Davidson's fixative, embedded and sectioned along the superior-inferior plane through the optic nerve, and as described in Ref. [14]. For light microscopic histologic analyses, sections were stained with hematoxylin (MHS16, Sigma, Burlington, MA, USA) and eosin (HT110116, Sigma). Following staining, the sections were mounted with permount mounting medium (SP15100, Fisher Scientific, Waltham, MA). Images were captured with 40Χ objective 500 μm away (both sides) from the optic nerve using a Zeiss Axioskop 50 (Carl Zeiss, White Plains, NY, USA). The thickness of the outer plexiform layer (OPL) and inner plexiform layer (IPL) was measured from the same sections at 500 μm distance from the optic nerve head in both superior and inferior regions with the line tool in ZEN 3.0 imaging software. For quantifications, photoreceptor nuclei were counted in 100 μm windows at intervals of 200 μm across the superior-inferior plane with ImageJ. The identity (RC vs. RDC groups) of the samples was masked to the observer who performed all morphometric measurements.

### 2.6 Immunoblotting

Frozen neural retinas were placed in 1Χ phosphate-buffered saline (PBS, pH 7.4) with 1 % Triton X-100 and protease inhibitors (Roche, Basel, Switzerland) and homogenized with a motorized pestle (VWR, Randor, PA) and 10 bursts of sonication. The mixture was incubated at 4 °C for an hour and later centrifuged at 4000×*g* for 10 min to separate the insoluble components. Thirty micrograms of supernatant were mixed with Laemmli buffer containing β-mercaptoethanol and incubated at room temperature for 10 min, then heated up at 95 °C for 5 min and loaded onto a 12 % SDS-PAGE. At the end of the run, proteins were transferred onto a PVDF membrane (Bio-Rad Laboratories, Hercules, CA) via standard protocols. Protein bands were detected with the specific primary antibodies (Table S1), and the membranes were visualized using a ChemiDoc™ Imaging system Version 3.0.1.14 (Bio-Rad). Quantitative analysis was done on unsaturated bands using Image Lab software version 6.1 (Bio-Rad). The intensity of these bands was then normalized to the intensity of the β-actin bands found within the same lanes of each immunoblot.

### 2.7 Immunofluorescence

Immunolabeling on 10 μm thick paraffin-embedded sections was performed as described previously [14,15]. Briefly, after rehydration and antigen retrieval (Tris-EDTA pH 9.0 with 0.1 % Tween), sections were washed in H_2_O, incubated in 1 % sodium borohydride for 3 min, washed in H_2_O, and incubated in blocking buffer (1 × PBS, pH 7.4, 2 % donkey serum, 0.5 % Triton X-100, and 50 mg/ml bovine serum albumin) for 1 h. After blocking, primary antibodies were applied in blocking buffer and incubated overnight at 4 °C. This was followed by three 10-min washes in 1 × PBS (pH 7.4), incubation with appropriate fluorescently labeled secondary antibodies in blocking buffer for 2 h, and an additional wash in 1x PBS. Sections were incubated in DAPI (Life Technologies, Carlsbad, CA) for 30 min. Slides were mounted with Prolong Gold antifade mountant (Thermo Fisher Scientific). Images in Fig. 3D were collected at an original magnification of 20 × and then tiled. Images in Fig. 4A were captured using a Zeiss LSM 800 confocal/Airyscan microscope with a 63 × oil objective (Carl Zeiss), represent a 5-slice stack, at 360 nm per slice, 1.76 μm total, and collapsed to a single projection image. Zen Image Analysis (Carl Zeiss) and Photoshop were utilized for processing the images. To maintain consistency and precision in the number of cones, sections that went through the center of the optic nerve were examined. For the generation of three-dimensional reconstruction, the 3D create tool in ZEN 3.2 software was used following adjustment of orientation in the YX or ZY plane and exported in TIFF format.

**Fig. 2:**
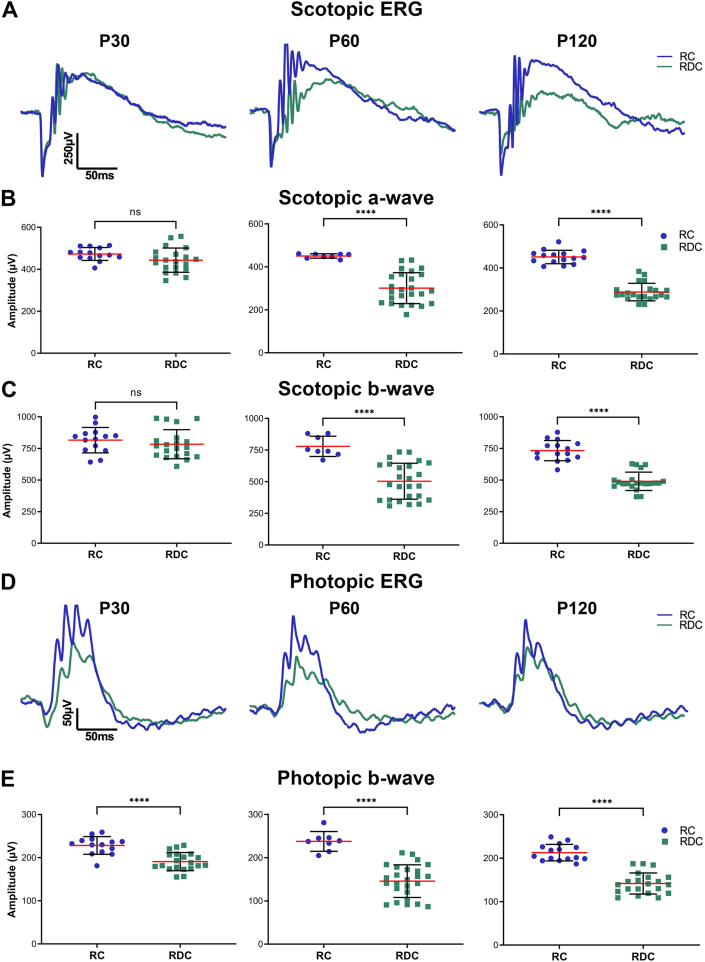
**Rod and cone functional decline in RDC-fed mice.** (**A**) Representative scotopic ERG waveforms from RC- and RDC-fed mice at P30, P60, and P120. (**B–C**) Scotopic a- (**B**) and b-wave (**C**) amplitudes measured at the indicated ages show a decrease in P60 and P120 RDC-fed mice compared to RC-fed controls. (**D**) Representative photopic ERG waveforms for RC and RDC-fed mice at P30, P60, and P120. (**E**) Photopic ERG amplitudes measured at the indicated ages reveal a reduction in P30 RDC-fed mice that did not progress through P120 when compared to RC-fed mice. Data are presented as mean ± SD. Statistical significance was determined using an unpaired *t*-test with the Mann-Whitney test. *P* values: *p* < 0.05(∗), *p* < 0.01(∗∗), *p* < 0.001(∗∗∗), *p* < 0.00001 (∗∗∗∗). Sample sizes (n values): Scotopic a-wave, scotopic b-wave, and photopic b-wave: RC P30 (14), P60 (8), P120 (15) and RDC P30 (20), P60 (25), P120 (21). (Each symbol represents the averaged value of both eyes from the animal).

**Fig. 3:**
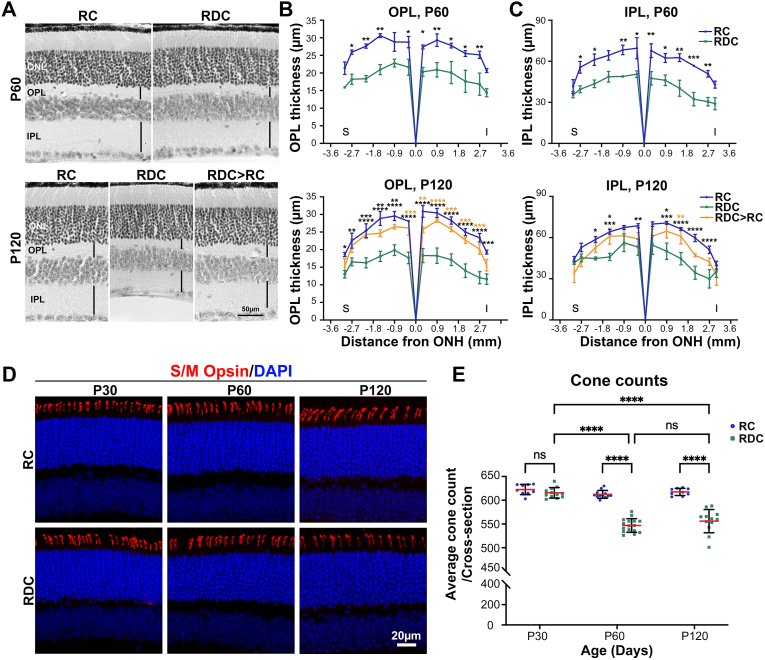
**Early ariboflavinosis leads to structural alterations and cone photoreceptor loss.** (**A**) Representative light microscopic images of retinal sections from RC- and RDC-fed mice at P60 (upper panel) and P120 (lower panel), including RDC > RC-fed mice at P120. (**B, C**) Thickness measurements of OPL (**B)** and IPL (**C**) show minimal changes at P60 but significant reductions at P120. Statistical comparisons were performed using two-way ANOVA with Sidak's multiple comparisons test for P60 and two-way ANOVA with Tukey's multiple comparisons test for P120 in (**B**) and (**C**). Data are presented as mean ± SEM. Sample sizes (n values): for OPL RC at P60 (3), P120 (3); for RDC at P60 (3), P120 (3); for RDC > RC P120 (3); for IPL RC at P60 (3), P120 (5); for RDC at P60 (3), P120 (3); and for RDC > RC at P120 (3). (**D**) Retinal cross-sections from RC- and RDC-fed mice labeled for S and M opsins at the indicated ages, 200 μm superior to the optic nerve head. *Scale bar:* 20 μm. Images were captured at 20x magnification. (**E**) Cone photoreceptor counts at different ages. Data are presented as mean ± SD. Statistical significance was determined by two-way ANOVA with Sidak's multiple comparisons test. Sample size (n values): Cone cells for RC at P30 (3), at P60 (3), at P120 (3); for RDC at P30 (4), at P60 (4), at P120 (4). *P* values: *p* < 0.05 (∗), *p* < 0.01 (∗∗), *p* < 0.001 (∗∗∗), *p* < 0.00001 (∗∗∗∗). IPL (inner plexiform layer), OPL (outer plexiform layer), ONL (outer nuclear layer), ONH (optic nerve head), S (superior), and I (inferior).

**Fig. 4:**
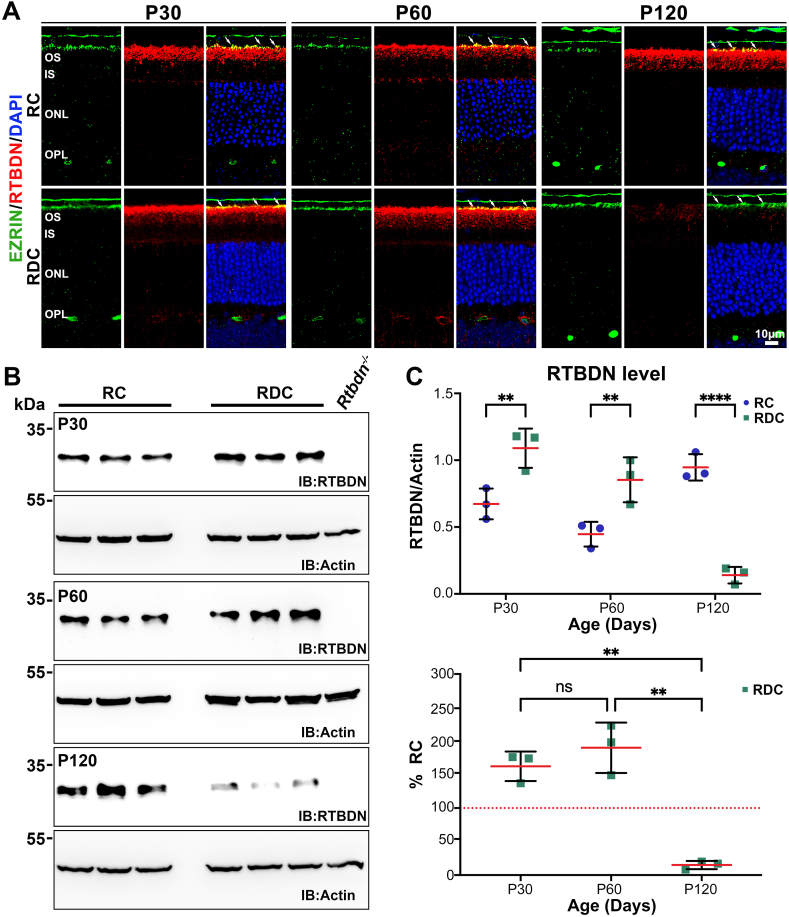
**Retbindin levels initially increased and then declined in RDC-fed mice.** (**A**) Immunofluorescence labeling of RTBDN (red) and EZRIN (green), with nuclei counterstained with DAPI (blue), in retinal sections from RC- and RDC-fed mice at P30, P60, and P120. OS (outer segments), IS (inner segments), ONL (outer nuclear layer), and OPL (outer plexiform layer). *Scale bars:* 10 μm. Images were captured at 63x magnification and represent collapsed planes from a confocal stack. (Arrows point to the interphase between RTBDN and EZRIN) (**B**) Representative immunoblots of RTBDN in neural retinas from RC- and RDC-fed mice at P30, P60, and P120. Samples from three independent retinas are presented. Immunoblot analysis shows an initial increase in RTBDN levels at early stages, followed by a sharp decline at P120 in RDC-fed mice (115 days on RDC diet). (**C**) Quantification of RTBDN levels relative to actin by immunoblotting from RC- and RDC-fed retinas at P30, P60, and P120 (upper panel), and RTBDN levels in RDC-fed mice expressed as a percentage of RC controls at indicated ages (lower panel). Data are presented are mean ± SD. Statistical significance was determined using two-way ANOVA with Sidak's multiple comparisons test (C, upper panel) and two-way ANOVA with Tukey's multiple comparisons test in (**C**, lower panel). Sample size (n values): RC at P30 (3), at P60 (3), at P120 (3), and RDC at P30 (3), at P60 (3), at P120 (3). *P* values: *p* < 0.05 (∗), *p* < 0.01 (∗∗), *p* < 0.001 (∗∗∗), *p* < 0.00001 (∗∗∗∗).

### 2.8 RPE flat-mount and cell count

Eyes were extracted from euthanized mice and placed in 4 % paraformaldehyde solution for 2 h. After the removal of the cornea and lens, the RPE was dissected away from the neural retina. The RPE was flat-mounted by making four incisions, washed twice in 1 × PBS (pH 7.4) for 10 min each, and blocked with blocking solution (5 % BSA, 2.5 % donkey serum, and 1 % Triton X-100 in 1 × PBS) for 2 h. The flattened eye cup was then incubated with anti-β-CATENIN antibody (Proteintech Group, Rosemont, Illinois) at 4 °C overnight. RPE was then washed four times with 1 × PBS for 10 min each and incubated with phalloidin (Thermo Fisher), secondary antibody, and DAPI for 2 h at room temperature. Following three washes with 1 × PBS, 10 min each, the RPE was mounted on a glass microscope slide. Images (40X, 160 μm × 160 μm) were obtained from a central location of each clover. Cells that were more than half positioned within the image frame were counted using ImageJ software and cell counts were graphed as per area of 0.0256 mm^2^ for each group.

### 2.9 Electroretinography

Full-field electroretinograms (ERG) were performed on dark-adapted animals at postnatal days (P30, P60, and P120) as described previously [15] using a UTAS system (LKC, Gaithersburg, MD). In brief, post-anesthesia, induced with a mixture of ketamine and xylazine, the pupil was dilated with 1 % cyclopentolate for 5 min. Subsequently, a single drop of Gonak (2.5 % Hypromellose) was applied, and a platinum wire loop electrode was placed on the corneal surface. Scotopic measurements were recorded in response to a single flash of white light at 157.7 cd-s/m^2^. After 5 min of light adaptation at 29.03 cd/m^2^, photopic ERGs were recorded and averaged from 25 flashes at 79 cd-s/m^2^. During the experiment, mice were kept on a 37^o^C platform to regulate their body temperature. Post-experiment, mice were observed until they had completely recovered from the effects of the anesthesia. ERG results obtained from both eyes were averaged for each animal and represented graphically using GraphPad Prism software version 9.5.1.

### 2.10 Statistical analysis

Statistical analyses were performed using GraphPad Prism 9.5.1. For comparisons between two groups, the unpaired *t*-test with Mann-Whitney test and the unpaired *t*-test with Welch's correction were used. For comparison between multiple groups, ordinary one-way ANOVA with Tukey's multiple comparison test, ordinary one-way ANOVA with Sidak's multiple comparisons test, two-way ANOVA with Tukey's multiple comparisons test, or two-way ANOVA with Sidak's multiple comparisons test was used.

## 3 Results

### 3.1 Riboflavin deficiency leads to a progressive decline in retinal function

To assess the impact of RF deficiency on retinal function, we conducted longitudinal recordings of light-evoked responses in RC- and RDC-fed mice. At P30 (25 days on RDC), scotopic a- and b-waves responses were comparable to those of age-matched RC controls (Fig. 2A–C, left panels). However, photopic ERG responses in P30 RDC mice were reduced by 16 % compared to RC controls (Fig. 2D and E, left panels). By P60, significant reductions were observed in scotopic a- (35.7 %) and b-waves (36.8 %) responses as well as in the photopic b-wave (36 %) responses in RDC mice, relative to age-matched RC-fed controls (Fig. 2A–E, middle panels). No further declines in scotopic a- and b- or photopic b-responses were detected by P120 (Fig. 2A–E, right panels).

### 3.2 Riboflavin deficiency induces structural changes in the retina

To assess the structural impact of RF deficiency on the retina, we measured the thickness of the outer plexiform layer (OPL) and inner plexiform layer (IPL) at P60 and P120 (Fig. 3A–C). We observed pan-retinal thinning in the OPL and IPL in RDC mice (Fig. 3A, black vertical lines). Statistically significant reductions in both OPL (∼30 %) and IPL (∼28 %) were observed at P60 in RDC mice (Fig. 3B and C, upper panels). At P120, RDC mice showed a reduction of ∼36 % in OPL and ∼30 % in IPL thicknesses (Fig. 3B and C, lower panels). To investigate whether the functional decline in cone responses at P30 is linked to cone cell loss, retinal sections from RC- and RDC-fed mice were immunostained for S/M opsin (Fig. 3D, S/M opsin, red) and cones number were counted in cross sections (Fig. 3E). Although we observed a decline in cone function at P30 (Fig. 2D and E), there was no reduction in cone numbers at this age in RDC mice (Fig. 3E). However, at P60, the number of cones in RDC mice was reduced by 10 % compared to RC mice, and no further decrease in cone numbers was observed at P120 (Fig. 3E).

To assess whether the structural and functional changes observed were associated with alterations in photoreceptor-specific proteins, we performed immunoblot analysis for rhodopsin (RHO), interphotoreceptor binding protein (IRBP, RBP3), arrestin (ARR1), peripherin 2 (PRPH2), and transducin (GNAT1). As shown in Fig. S1A and quantified in Fig. S1B, protein levels were comparable between RC and RDC retinas. To relate these findings to the late-stage RDC model [21], we investigated the levels of transcripts of the same photoreceptor proteins. As shown in Fig. S1C, transcripts for *Rho, Rbp3, Arr1, Prph2*, and *Gnat1* were the same in RC and RDC retinas.

### 3.3 Riboflavin deficiency alters RTBDN and flavins levels

Our previous study identified RTBDN as a retinal-specific RF binding protein [14], which helps maintain retinal flavin levels and plays a protective role in various retinal degeneration models [16,31]. In the nutritional model of adult-onset ariboflavinosis, where mice were fed RDC starting at P30, we found that RTBDN levels were reduced as long as the mice remained on the RDC diet, but these levels were restored upon RF supplementation [21]. To further explore the relationship between RTBDN and RF during postnatal development upon RF elimination, we labeled retinal sections for RTBDN collected from RDC mice at P30, P60, and P120 (Fig. 4A–red). We also labeled for the RPE microvilli marker, EZRIN (Fig. 4A, green). At both P30 and P60, the RTBDN expression pattern in RDC mice was similar to that in RC mice, primarily localized around photoreceptor outer segments (OSs), with a focus at the tip (white arrows, Fig. 4A) [14,15]. However, by P120, RTBDN labeling was significantly reduced in retinas from RDC mice (115 days on RDC) compared to RC controls (Fig. 4A). To quantify the changes in RTBDN levels at these three timepoints, we performed immunoblots on retinal extracts. At P30 and P60, RTBDN levels in RDC retina were significantly higher compared to those in RC retina (Fig. 4B and C). Interestingly, at P120, RTBDN levels in the RDC retina were reduced by 85 % compared to RC retina (Fig. 4B and C).

**Fig. 5:**
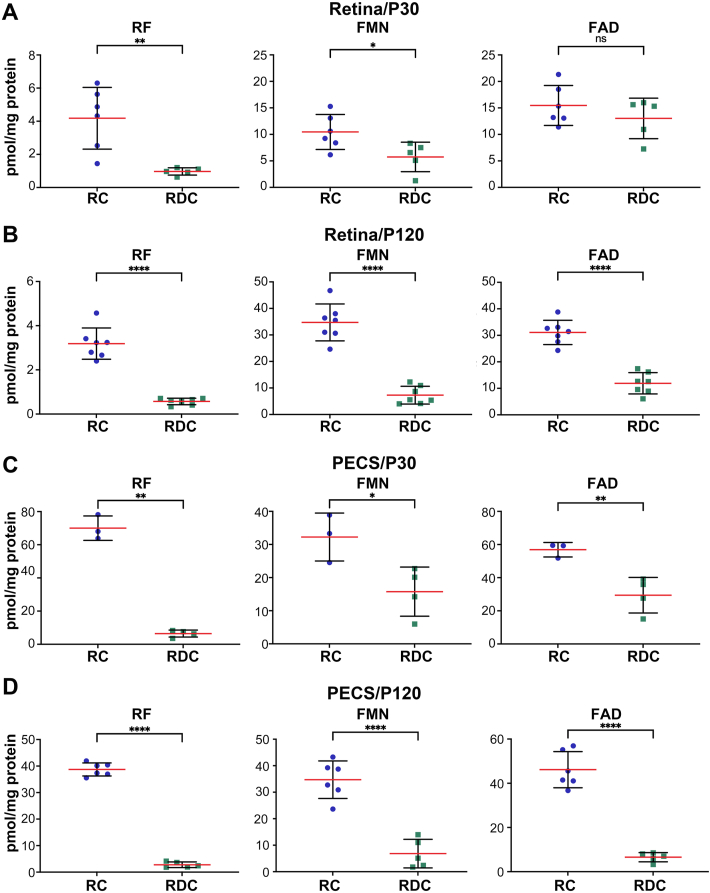
**Flavin levels in the retina and PECS are reduced in RDC-fed mice.** (**A, B**) Levels of RF, FMN and FAD in the retina of RC- and RDC-fed mice at P30 **(A)**, P120 **(B)**. (**C, D**) Levels of RF, FMN, and FAD in the PECS of RC- and RDC-fed mice at P30 (**C**) and P120 PECS (**D**). Data are presented are mean ± SD. Statistical significance was determined using an unpaired *t*-test with Welch's correction. Sample size (n values): Retina from RC at P30 (6), at P120 (7) and from RDC at P30 (5), P120 (7), and PECS from RC at P30 (3), at P120 (6), and from RDC at P30 (4) and at P120 (5). *P* values: *p* < 0.05 (∗), *p* < 0.01 (∗∗), *p* < 0.001 (∗∗∗), *p* < 0.00001 (∗∗∗∗).

We next evaluated the levels of RF and its cofactors, FAD and FMN, in extracts from the retinas and PECSs of RC and RDC at P30 and P120 using our previously optimized HPLC method [29]. At P30, we observed a significant reduction in RF (∼77 %) and FMN (∼45 %) levels in the retina of RDC mice in comparison to RC mice, while FAD levels showed a non-significant decrease (Fig. 5A). By P120, RF was reduced by 82 %, FMN by 79 % and FAD by 62 % compared to RC retinas (Fig. 5B). In the PECS, flavins levels began to decline significantly by P30 and were almost depleted by P120 (Fig. 5C and D). Although at P30, its levels in the RDC retina remained similar to those in the RC retina, PECS's FAD level was reduced by 48 % (Fig. 5A and C). By P120, RF, FAD, and FMN in both retinas and PECSs followed similar patterns of decline, with FAD being preserved slightly longer in the retina of RDC mice.

### 3.4 Structural changes in the RPE associated with feeding on RDC

**Fig. 6:**
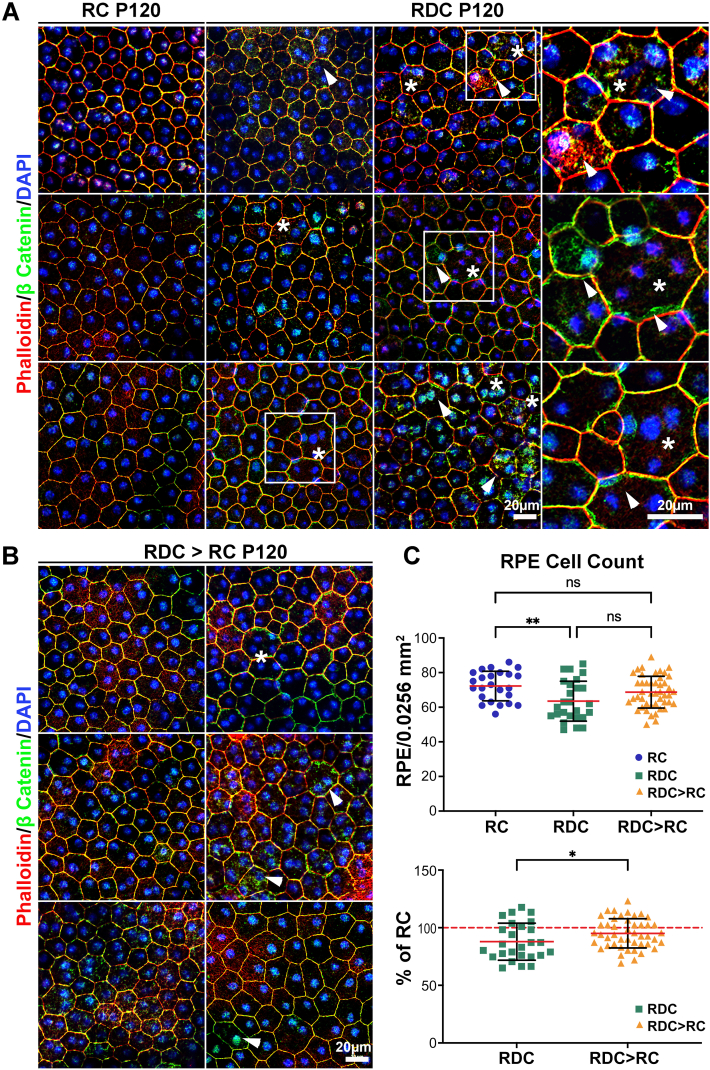
**Riboflavin deficiency induces RPE dystrophy that is partially rescued by RF supplementation.** (**A**) RPE flat-mounts from P120 RDC-fed mice stained for β-CATENIN (green) and phalloidin (red), showing significant intracellular localization of β-CATENIN (arrowheads) away from the plasma membrane and the presence of multinucleated, enlarged RPE cells (asterisks) compared to RC-fed mice. Boxes highlight the areas that are on the far right. (**B**) RPE flat-mounts from RDC > RC stained for β-CATENIN (green) and phalloidin (red). These flat-mounts show reduced cytoplasmic accumulation of β-CATENIN (arrowheads) and a lower number of multinucleated RPE cells (asterisks) compared to RDC-fed mice. **C.** Quantification of RPE cell numbers from flat-mount images (upper panel). RPE cell counts were normalized to RC controls and plotted as a percentage for RDC and RDC > RC groups (lower panel). Images in (**A**) and (**B**) were captured at 40x magnification and represent collapsed planes from a confocal stack. *Scale bars:* 20 μm. Each experiment was performed three times using independent samples. RPE cell numbers were quantified from 6 to 16 regions within a 0.0256 mm^2^ window in 3–4 independent samples per cohort. Data are presented as mean ± SD. Statistical significance was determined using ordinary one-way ANOVA with Tukey's multiple comparison test for (C, upper panel) and unpaired *t*-test with Mann-Whitney test for (**C**, lower panel). *P* values: *p* < 0.05 (∗), *p* < 0.01 (∗∗), *p* < 0.001 (∗∗∗), *p* < 0.00001 (∗∗∗∗). Sample size (n values): for RC (4), RDC (3), and RDC > RC (3).

We have previously demonstrated that mice that were placed on the RDC diet at P30 exhibited an increase in multinucleated RPE cells at P300 [21], which is a common phenomenon of various RPE pathologies [32,33]. To investigate the changes when diet started during postnatal development, we examined the RPE structure at P120, a time point when the functional decline remained unchanged from P60 (Fig. 2A–E). Under normal conditions, the RPE cells form a regular, hexagonal pattern (Fig. 6A, left panels). However, in the RDC mice, we observed loss of the hexagonal shape and an increase in multinucleated RPE cells (Fig. 6A, asterisks in right panels), a phenotype commonly associated with RPE atrophy [32], and evident across multiple regions of the RPE flat mounts (Fig. S2A–D). To quantify the observed changes, we counted RPE cells and observed a ∼12 % reduction in the average number of cells per field compared to RC controls (Fig. 6C). However, we observed a statistically significant increase in the number of cells that contained more than two nuclei (Fig. S2D). Moreover, RPE flat mounts collected at P120 from RDC mice showed intracellular localization of β-CATENIN (Fig. 6A and B, green, arrowheads) away from the plasma membrane. There was a significant increase in the number of RPE cells with mislocalized β-CATENIN in RDC animals (Fig. S2D). We also observed cells that harbored more than two nuclei (Fig. 6A and B, asterisks).

### 3.5 Impacts of riboflavin deficiency on other vitamins

Flavins, as essential cofactors, play major roles in the activation and synthesis of other vitamins. FMN serves as a cofactor for pyridoxine (pyridoxamine) phosphate oxidase, the enzyme responsible for converting pyridoxine (vitamin B6) into its active form, pyridoxal 5′-phosphate (PLP) [1,4,10,[34], [35], [36]]. Additionally, both FAD and PLP are required cofactors in the conversion of tryptophan to niacin (vitamin B3) [36]. Niacin is the precursor of the coenzymes nicotinamide adenine dinucleotide (NAD^+^) and nicotinamide adenine dinucleotide phosphate (NADP^+^), which are involved in numerous metabolic processes [36,37]. Furthermore, the activation of thiamine (vitamin B1) to thiamine pyrophosphate (TPP) requires a FAD-dependent enzyme [1,38,39].

To assess the impact of RF deficiency on these vitamin-derived cofactors, we examined their levels using metabolomic analysis in the late-stage RF deficiency model [21]. In the retina, levels of TPP and PLP remained unchanged; however, nicotinamide levels were significantly reduced (Fig. S3A). In contrast, both TPP and nicotinamide levels were significantly decreased in the PECS of RDC animals (Fig. S3B). Although nicotinamide is the precursor for NAD^+^ and NADP^+^, the levels of these two coenzymes were not altered in the PECS. Interestingly, despite the reduction in nicotinamide, levels of NADH, the reduced form of NAD^+^, were unexpectedly elevated in the retina (Fig. S3C and D).

### 3.6 RF supplementation rescues some of the phenotypes caused by its deficiency

**Fig. 7:**
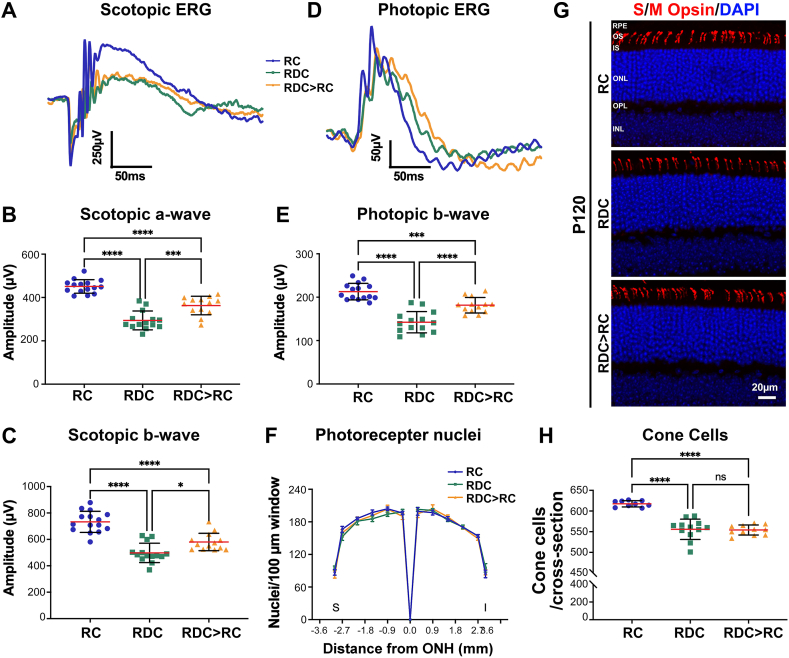
**Sustained riboflavin supplementation partially rescues retinal function and RPE degeneration.** (**A**) Representative scotopic ERG waveforms from P120 RC-, RDC-, and RDC > RC-fed mice. (**B**, **C**) Partial recovery of scotopic-a wave (**B**) and b-wave (**C**) responses in the RDC > RC group. (**D**) Representative photopic ERG waveforms from P120 RC-, RDC-, and RDC > RC-fed mice. (**E**) Improvement in photopic b-wave responses observed in the RDC > RC group. Data are presented as mean ± SD. Statistical significance was determined using ordinary one-way ANOVA with Tukey's multiple comparisons test. Sample size (*n* values): Scotopic a-wave, scotopic b-wave, and photopic b-wave: RC (15), RDC (14), and RDC > RC (13). (**F**) Photoreceptor counts from representative light microscopic images of P120 RC-, RDC-, and RDC > RC-fed mice, showing no change in photoreceptor numbers. Data are presented as mean ± SEM. Statistical significance was determined using two-way ANOVA with Sidak's multiple comparison test. Sample size (*n* values): RC (3), RDC (3), and RDC > RC (3). (**G**) Representative images of retinal cross sections from P120 RC-, RDC-, and RDC > RC-fed mice labeled for S and M opsin (red), taken at 200 μm superior to the optic nerve head. *Scale bar:* 20 μm. Images captured at 20x magnification. (**H**) Quantification of cone cells at P120 across groups shows that RF supplementation does not rescue cone loss. Data presented as mean ± SD. Statistical significance was determined using ordinary one-way ANOVA with Sidak's multiple comparisons test. Sample size (*n* values): at P120 from RC (3), RDC (4), and RDC > RC (4). (each symbol represents one count). *P* values: *p* < 0.05 (∗), *p* < 0.01 (∗∗), *p* < 0.001 (∗∗∗), *p* < 0.00001 (∗∗∗∗).

Ariboflavinosis resulting from mutations in *SLC52A2* can be treated with high oral doses of RF supplementation that improve neurodegenerative symptoms [2,6,23,27]. In our previous study on RF deficiency starting at P30, we found that RF supplementation at P120 restored total retinal flavins and RTBDN protein levels, leading to functional and structural improvements in the neural retina and the RPE [21]. To assess whether RF supplementation could also rescue the retina and RPE defects in our developmental ariboflavinosis model, we switched a group of mice from RDC to RC (RDC > RC) at P60 and analyzed them at P120 (Fig. 1). After 60 days on RC diet, RDC > RC mice showed improved retinal function compared to RDC mice (Fig. 7A–E), with significant increases in scotopic a-wave (23 %), scotopic b-wave (17 %) and photopic b-wave (27 %) (Fig. 7A–E). However, the rescue was incomplete compared to RC mice. Structurally, RDC > RC mice showed significant rescue of OPL and IPL thicknesses (Fig. 3B and C, lower panel, orange lines). The number of nuclei in the ONL remained unchanged (Fig. 7F). Despite partial functional recovery in cones, RF supplementation did not increase cone numbers in RDC mice (Fig. 7G and H).

**Fig. 8:**
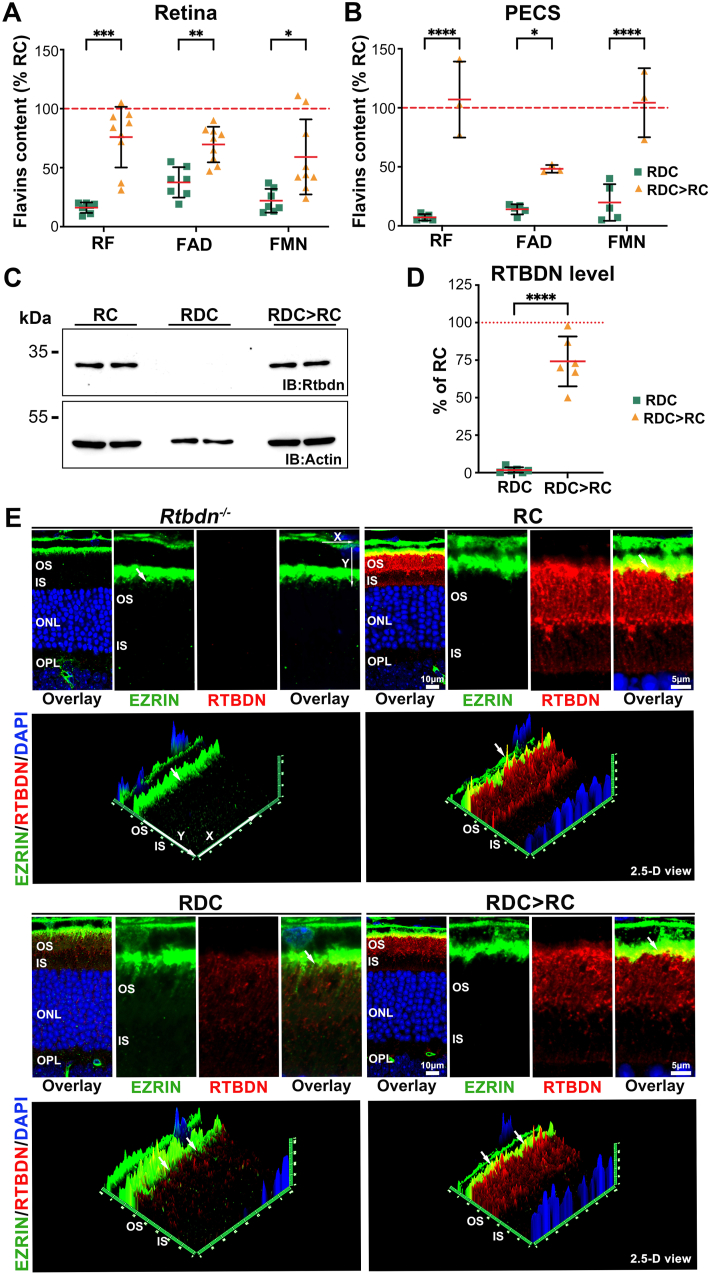
**Recovery of flavins and RTBDN protein levels following RF supplementation.** (**A, B**) Flavin levels in the retina (**A**) and PECS (**B**) recovered to normal levels by P120 in mice that received RF supplementation starting at P60. Flavin levels are expressed as a percentage of those in RC-fed P120 mice. Data are presented as mean ± SD. Statistical significance was determined using two-way ANOVA with Sidak's multiple comparisons test. Sample size (*n* values): Retina: RDC (7), RDC > RC (9), and PECS: RDC (5), RDC > RC (3). (**C**) Representative immunoblot of two independent retinal extracts from P120 RC-, RDC-, and RDC > RC-fed mice. (**D**) Quantification from three independent experiments showing the reappearance of RTBDN following RF reintroduction (RDC > RC). Data are presented as mean ± SD. Statistical significance was determined using an unpaired *t*-test. *P* values: *p* < 0.05 (∗), *p* < 0.01 (∗∗), *p* < 0.001 (∗∗∗), *p* < 0.00001 (∗∗∗∗). (**E**) Immunofluorescence labeling of Rtbdn (red) and EZRIN labeling (green), with nuclei counterstained with DAPI (blue), in retinal sections from P120 RC-, RDC-, and RDC > RC-fed mice. The 2.5-D reconstruction (lower panels) illustrates the spatial distribution and signal intensity of labeling with x- and y-coordinates indicating the anticlockwise rotation of the image, and intensity shown on the *z*-axis. Images in (**E**) were captured at 63x magnification and represent collapsed planes from a confocal stack (1.76 μm). OS (outer segments), IS (inner segments), ONL (outer nuclear layer), and OPL (outer plexiform layer).

To assess flavin levels, we performed HPLC on the retina and PECS (Fig. 8A and B). In RDC > RC mice, retinal flavin levels showed a ∼69 % recovery but did not fully reach RC levels (Fig. 8A). In PECS, RF and FMN recovered to RC levels, while FAD remained lower (Fig. 8B). This flavin recovery correlated with an increase in RTBDN levels, reaching 74 % of those in RC retinas (Fig. 8C and D), with proper localization confirmed by IHC (Fig. 8E, white arrow).

Some RPE phenotypes associated with RF deficiency showed partial improvement in RDC > RC-fed mice (Fig. 6B and C). By P120, RDC mice exhibited a significant reduction in RPE cell number compared to RC-fed mice (Fig. 6C). In RDC > RC, RPE cell number improved by 10 % compared to RDC mice with marked improvement in the hexagonal shape of the cells (Fig. 6C). Although the number of cells with cytoplasmic β-CATENIN accumulation improved, there remained a statistically significant number of cells with the mislocalized β-CATENIN in RDC > RC animals (Fig. S2D), and while the number of enlarged, multinucleated RPE cells decreased, they were still present (Fig. 6A and B, asterisks). This suggests that some changes may be irreversible or that a longer duration of RF supplementation is required for full recovery.

## 4 Discussion

In our previous study on an adult-onset ariboflavinosis model, RF deficiency led to decreased retinal function, structural changes in the retina and RPE, and abnormalities in retinal metabolic activity [21]. To investigate the visual complications associated with RF deficiency during retinal development, we established a nutritional mouse model of early-onset ariboflavinosis.

Herein, we found that early-onset ariboflavinosis led to both scotopic and photopic functional decline, accompanied by cone photoreceptor loss, whereas rod photoreceptors remained unaffected. This contrasts with our previous findings in the adult-onset model, where RF deficiency primarily affected rods at later stages but had no impact on cones. [21]. These results suggest that cone development may be particularly dependent upon flavins, as no further reduction in cone photoreceptor numbers was observed beyond P60. This is significant, as human ariboflavinosis results from impaired RF transporter function early in embryonic development, and the role cones play in macular structure and function. Our findings have important implications for understanding how RF deficiency contributes to early-onset retinal diseases, particularly in at-risk populations such as infants and malnourished individuals [2,3,6].

An intriguing finding in this study is the biphasic response of RTBDN to early ariboflavinosis. At P30 and P60, RTBDN levels were upregulated, suggesting a protective mechanism in which RTBDN sequesters flavins in the retina, as observed in our previous studies [16,31]. However, the subsequent decrease in RTBDN levels implies that its stability depends on flavin binding, and in their absence, RTBDN undergoes degradation. This is further supported by our observation in both current and adult-onset models, where RTBDN levels returned to normal following RF supplementation [21].

In the current model of RF deficiency, we observed a reduction in ERG responses without a corresponding decrease in the number of photoreceptors, as indicated by the preserved thickness and nuclei count in the outer nuclear layer. That is in agreement with our previous observations in the late-onset RF deficiency model. We also showed that the thickness of both the inner and outer plexiform layers is the major change. Furthermore, RF supplementation led to a partial but statistically significant recovery of ERG responses. We propose that the incomplete functional recovery reflects only a partial restoration of the structural integrity of both the inner and the outer plexiform layers, underscoring the importance of these layers in mediating retinal function.

We also found that RF deficiency leads to significant RPE dystrophy, similar to the effects observed in adult-onset ariboflavinosis [21]. The RPE, which plays a crucial role in supporting photoreceptor function and maintaining retinal health [40,41], exhibited structural disruptions under RF-deficiency. Specifically, we observed a loss of the hexagonal shape of RPE cells and an increase in multinucleated cells. These changes likely stem from RPE cell fusion, possibly as a mechanism to reduce metabolic demands. This hypothesis is supported by the partial recovery in RPE cell numbers following RF supplementation. However, whether RPE cell numbers can fully recover to baseline levels remains an open question, requiring longer-term supplementation studies. The impact of ariboflavinosis on the RPE, whether occurring early or late, underscores the high sensitivity of these cells to RF deficiency. Nonetheless, the observed partial recovery in retinal and RPE functions and structures highlights the therapeutic potential of RF supplementation in mitigating or even reversing some associated visual impairments.

A key limitation of this study is the use of a single animal model, which may restrict the generalizability of our findings to human physiology. While the mouse model offers valuable insights into the effects of RF deficiency on retinal development, there are inherent differences between rodent and human retinas that may influence how RF deficiency manifests in humans.

In conclusion, this study underscores the critical role of RF in maintaining retinal and RPE health, particularly during early development. We demonstrated that early-onset ariboflavinosis leads to significant cone degeneration and RPE dystrophy, accompanied by a severe decline in RF, FMN, and FAD levels. Notably, RF supplementation effectively restored normal RF, FMN, and FAD levels while partially rescuing rod function and RPE structure. However, cone numbers and function were not restored, which underscores the importance of flavins for the development of the cones. Our findings draw important parallels with RF transporter deficiency disease (RTD), a neurological disorder caused by mutations in *SLC52A2* (RTD2) or *SLC52A3* (RTD3) that manifests in infancy [3,20,23,27]. The role of flavins in cone development is of significance to RTD patients, specifically for the development of macular cones. Given that high-dose RF therapy has been shown to improve RTD clinical symptoms [2,5,6,22,23,27], RF supplementation may also have the potential to rescue some retinal and RPE function in RTD patients. This study highlights the essential role of RF in retinal development and its potential as a therapeutic strategy for preventing or reversing retinal deficiencies in RTD. Further research is needed to investigate the long-term effects of RF supplementation and the applicability of the findings to human retinal disease.

## 5 Conclusions

Early onset riboflavin deficiency initially impairs cone photoreceptor function, followed by rod photoreceptor dysfunction. The decline in cone function results from a reduced cone photoreceptor number. Additional retinal changes include thinning of outer and inner plexiform layers, alongside structural alterations in the retinal pigment epithelium, characterized by multinucleated cells and loss of cellular hexagonal morphology. These functional and structural changes coincide with the depletion of retbindin. Riboflavin supplementation partially mitigates these phenotypic effects of deficiency. These findings underscore the critical importance of early identification of patients with mutations in riboflavin transporters and prompt riboflavin intervention in managing this condition. Understanding the precise effects of riboflavin deficiency on retinal structure and function provides valuable insights for developing targeted therapies to prevent or reverse vision loss.

## CRediT authorship contribution statement

**Xue Zhao:** Writing – original draft, Validation, Methodology, Investigation, Formal analysis, Data curation, Conceptualization. **Mustafa S. Makia:** Visualization, Formal analysis, Data curation. **Muna I. Naash:** Writing – review & editing, Supervision, Resources, Project administration, Investigation, Funding acquisition, Conceptualization. **Muayyad R. Al-Ubaidi:** Writing – review & editing, Supervision, Resources, Project administration, Investigation, Funding acquisition, Conceptualization.

## Declaration of generative AI in scientific writing

Neither AI nor AI-assisted technologies were used in the design, implementation, or writing of this manuscript.

## Funding sources

This work was supported by grants from the 10.13039/100000002NIH/10.13039/100000053NEI [EY010609 (MIN and 10.13039/100005190MRA), EY034671 (MIN), EY033872 (MIN), and the 10.13039/100001116Foundation Fighting Blindness (10.13039/100005190MRA). NIH had no role in the collection, analysis, or interpretation of the data; in the writing of the manuscript; or in the decision to submit the manuscript for publication.

## Declaration of competing interest

Authors declare no conflict of interests.

## Data Availability

All data are presented in the manuscript.
