# Supplementary material for: Developmental riboflavin deficiency results in structural and functional changes in the neural retina and RPE
Source: Redox Biol. 2025 Jul 16;85:103772. doi: 10.1016/j.redox.2025.103772 (PMC12301974; doi:10.1016/j.redox.2025.103772)
Supplement: Multimedia component 1 [file mmc1.pdf]

Supplementary Information

Antigen	Species	Clone	Application/Concentration	Source
Rtbdn	Mouse	6B6H5E11	1:10 (WB)	In house
Rtbdn	Rabbit		1:250 (IF)	Cloud-Clone Corp Cat# PAH203Mu01
Ezrin	Mouse	3C12	1:500 (IF)	ThermoFisher Cat# 35-7300
S-Opsin	Rabbit		1:250 (IF)	Sigma-Aldrich Cat# AB5407, RRID:AB_177457
M-Opsin	Rabbit		1:250 (IF)	Sigma-Aldrich Cat# AB5405, RRID:AB_177456
β Catenin	Rabbit		1:500 (IF)	Protein Tech Cat#51067-2-AP RRID: AB_2086128
Phalloidin Alexa Fluor 647			1:1000 (IF)	ThermoFisher Cat# A22287 RRID: AB_2620155
Actin-HRP	Mouse	AC-15	1:10,000 (WB)	Sigma-Aldrich Cat# A3854, RRID:AB_262011
Mouse IgG HRP	Donkey		1:10,000 (WB)	Sigma-Aldrich Cat# AP130P
Rabbit IgG Alexa Fluor 488	Donkey		1:1000 (IF)	ThermoFisher Cat# A21206, RRID:AB_2535792
Rabbit IgG Alexa Fluor 555	Goat		1:1000 (IF)	ThermoFisher Cat# A21428, RRID:AB_2535849
Rabbit IgG Alexa Fluor 647	Donkey		1:1000 (IF)	ThermoFisher Cat# A31573, RRID:AB_2536183
Mouse IgG Alexa Flour 488	Donkey		1:1,000 (IF)	ThermoFisher Cat# A21202, RRID:AB_141607
Mouse IgG Alexa Flour 647	Donkey		1:1,000 (IF)	ThermoFisher Cat# A-32787, RRID:AB_2762830
DAPI (stain)			1:1,000 (IF)	ThermoFisher Cat# 62248

Table S1. List of antibodies used in the current study

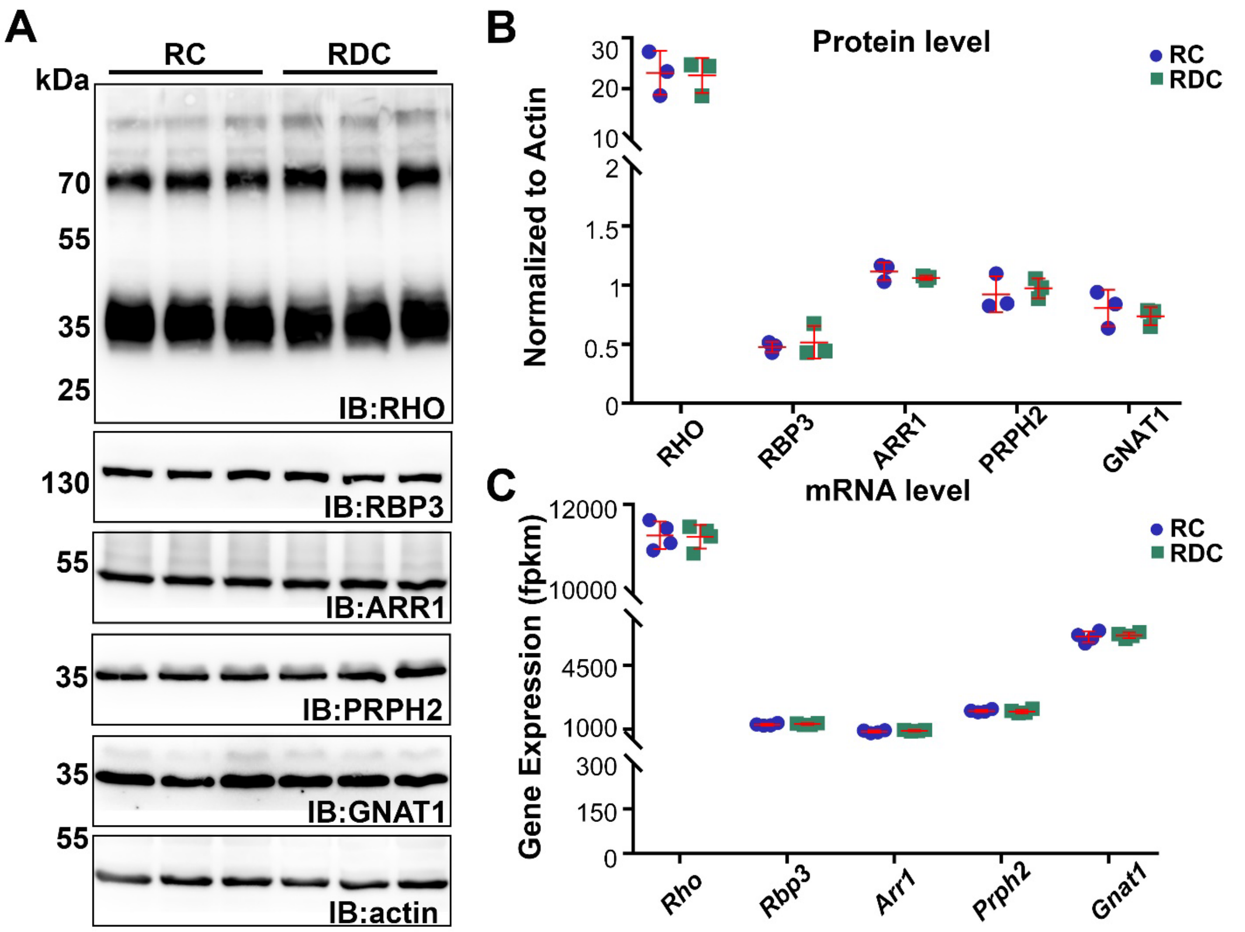

Figure S1. Expression of different photoreceptor-specific genes and proteins. (**A**) Representative immunoblots showing a pattern of expression of different retinal proteins from RC and RDC mice at P120. Samples from three independent retinas are presented. Blots were subsequently probed with anti-Rhodopsin, RBP3 (aka, IRBP, interphotoreceptor retinoid binding protein), Arrestin, PRPH2, Transducin, and β-actin. (**B**) Quantification of protein levels from three independent immunoblots relative to actin. Data are presented are mean ± SD. Statistical significance was determined using a multiple t-test. Sample size (n values): P120 RC (3) and RDC (3). (**C**) Transcript levels for proteins presented in **B** in P120 RDC and RC retinas from mice placed on the diet at P30. Expression levels were measured in FPKM (Fragments per kilobase of transcript per million mapped reads) from RC (purple) and RDC (green). Data are presented are mean ± SD. Statistical significance was determined using two-way ANOVA with Sidak's multiple comparisons test. Sample size (n values): P120 RC (4) and RDC (4). P values: $p<0.05$ (*), $p<0.01$ (**), $p<0.001$ (***), $p < 0.00001$ (****).

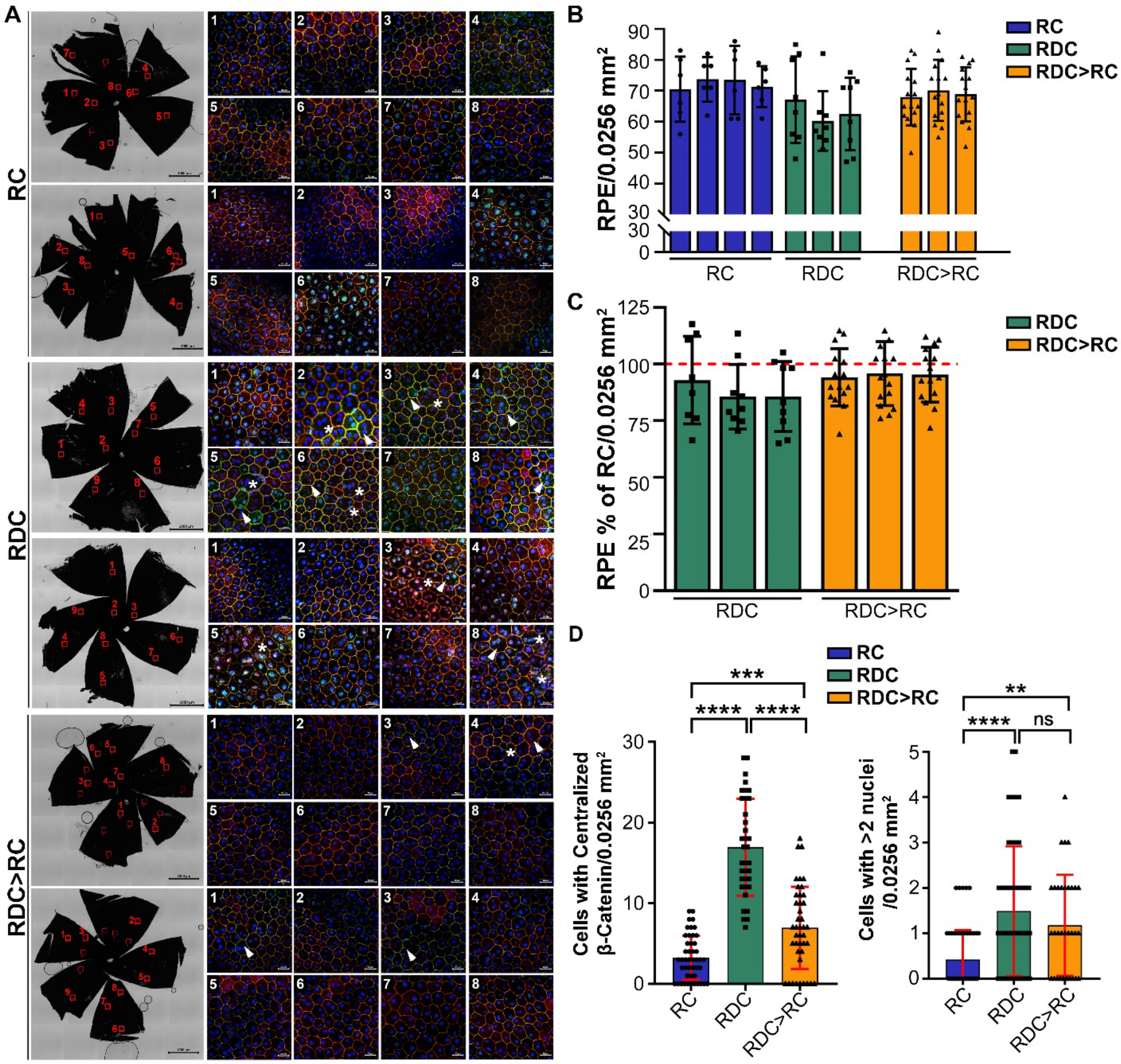

Figure S2. Riboflavin deficiency affects RPE cell structure and function, and is partially rescued by riboflavin supplementation. (**A**) Animals were divided into three groups: 1) RC fed until P120, 2) RDC fed until P120, and 3) RDC fed until P60, then switched to RC until P120 (RDC>RC). Representative eight regions were selected randomly in each RPE flat mount (marked by red boxes and numbers) and are shown on the right with their respective numbers. Two independent experiments are shown for each group. **B**) RPE cells were counted and plotted from every individual red box in (**A**). (**C**) RPE cell numbers per individual red box were quantified and plotted as a percentage of RC from RDC and RDC>RC groups. (**D**) RPE cell numbers with intracellular accumulation of β-catenin (left) and RPE cell numbers with more than 2 nuclei (right)

were quantified from 6-16 regions in 0.0256 mm^2 windows in 3-4 independent samples from RC, RDC and RDC>RC groups. Irregular-shaped large cells with multi-nuclei along with significant intracellular mislocalization of β-catenin, indicating significant dystrophy as seen in RDC fed animals, while these abnormalities are partially rescued in animals returned to RC for 60 days. Images in (**A**) were captured at magnification ×20 (overview images) ×40 (regional images) and shown are collapsed planes from a confocal stack. *Scale bars:* 100 µm (overview images) and 20 µm (regional images). Data are presented are mean ± SD. Statistical significance was determined using ordinary one-way ANOVA with Turkey's multiple comparisons test. *P* values: $p<0.05$ (*), $p<0.01$ (**), $p<0.001$ (***), $p < 0.00001$ (****). Sample size (n values): RC (4), RDC (3) and RDC>RC (3).

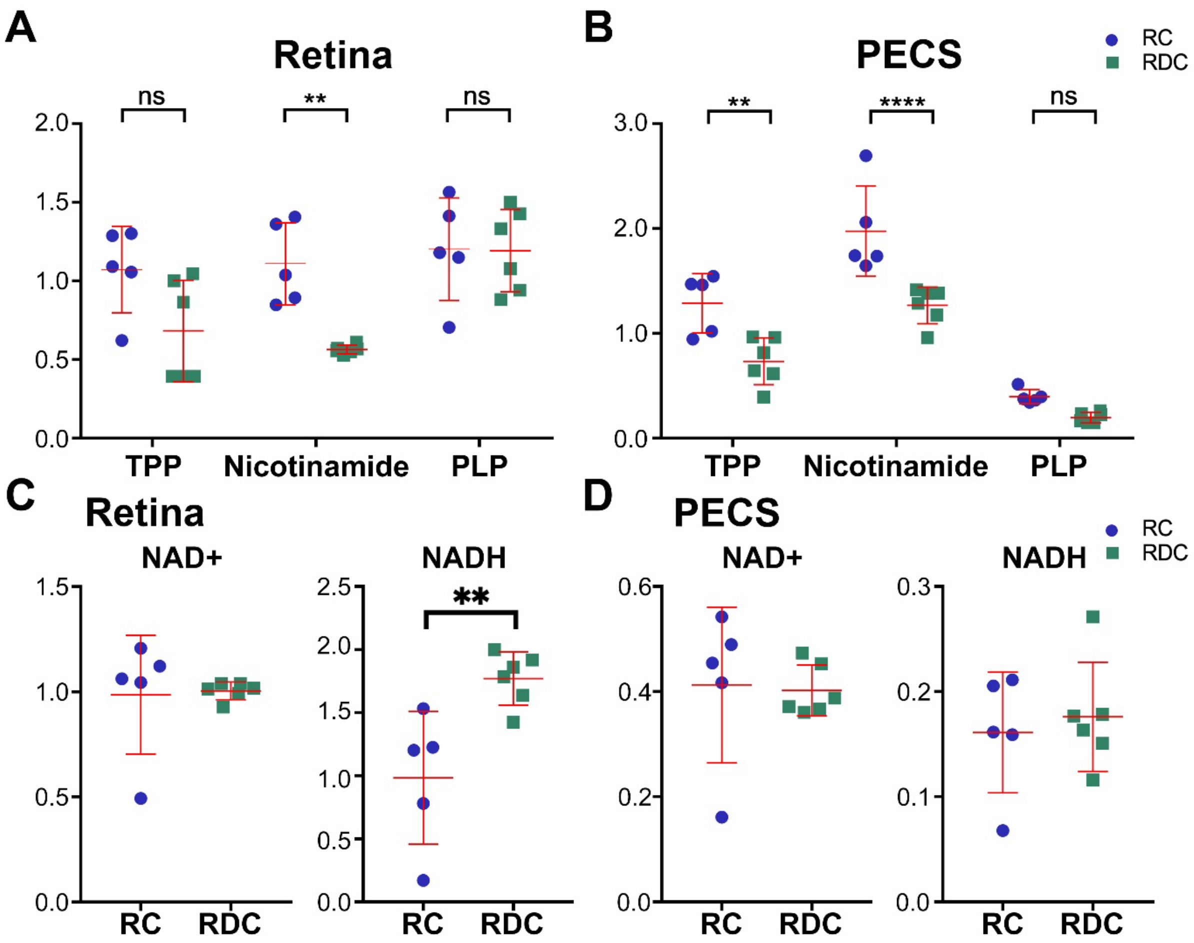

Figure S3. Effect of RDC on other important cellular factors. Levels of thiamine pyrophosphate (TPP), nicotinamide, and pyridoxal phosphate (PLP) in the retina (**A**) and PECS (**B**) from P120 RC- and RDC-fed mice. Statistical significance was determined using two-way ANOVA with Sidak's multiple comparisons test. Sample size (n values): Retina and PECS from P120 RC (5) and RDC (6). NAD and NADH levels in the retina (**C**) and PECS (**D**) from P120 RC- and RDC-fed mice. Statistical significance was determined using an unpaired t-test with Mann Mann-Whitney test. Sample size (n values): Retina and PECS from P120 RC (5) and RDC (6). P values: p<0.05 (*), p<0.01 (**), p<0.001 (***), p < 0.00001 (****).
